# Ophiobolin A Induces Autophagy and Activates the Mitochondrial Pathway of Apoptosis in Human Melanoma Cells

**DOI:** 10.1371/journal.pone.0167672

**Published:** 2016-12-09

**Authors:** Carlo Rodolfo, Mariapina Rocco, Lucia Cattaneo, Maria Tartaglia, Mauro Sassi, Patrizia Aducci, Andrea Scaloni, Lorenzo Camoni, Mauro Marra

**Affiliations:** 1 Department of Biology, University of Rome Tor Vergata, Rome, Italy; 2 Department of Science and Technology, University of Sannio, Benevento, Italy; 3 Proteomics & Mass Spectrometry Laboratory, ISPAAM-National Research Council, Naples, Italy; IDI, Istituto Dermopatico dell'Immacolata, ITALY

## Abstract

Ophiobolin A, a fungal toxin from *Bipolaris* species known to affect different cellular processes in plants, has recently been shown to have anti-cancer activity in mammalian cells. In the present study, we investigated the anti-proliferative effect of Ophiobolin A on human melanoma A375 and CHL-1 cell lines. This cellular model was chosen because of the incidence of melanoma malignant tumor on human population and its resistance to chemical treatments. Ophyobolin A strongly reduced cell viability of melanoma cells by affecting mitochondrial functionality. The toxin induced depolarization of mitochondrial membrane potential, reactive oxygen species production and mitochondrial network fragmentation, leading to autophagy induction and ultimately resulting in cell death by activation of the mitochondrial pathway of apoptosis. Finally, a comparative proteomic investigation on A375 cells allowed to identify several Ophiobolin A down-regulated proteins, which are involved in fundamental processes for cell homeostasis and viability.

## Introduction

Ophiobolins (OPs) are a family of sesterpenoid phytotoxic metabolites produced mainly by fungi of the *Bipolaris* genus, pathogen of rice, maize and sorghum [1,2]. In plants, OPs induce a wide array of toxic effects, such as inhibition of coleoptiles and root growth and seed germination inhibition [2]. At the cellular level, OPs affect membrane permeability, causing electrolytes and nutrients leakage, proton extrusion inhibition, decrease of CO_2_ photosynthetic assimilation and inhibition of protein and nucleic acid synthesis [2].

In the last years, accumulating evidence demonstrates that OPs are able to affect cellular functions also in mammalian cells. Ophiobolin A (OP-A) results strongly cytotoxic to mouse leukaemia cells, where it induces shrinkage of cell soma, chromatin condensation and DNA laddering [3], typical features of apoptotic cell death. Moreover, ophiobolin O (OP-O) from *Aspergillus ustus* induces apoptosis in multidrug-resistant MCF-7 breast cancer cells. [4,5].

By contrast, OP-A displays the same cytostatic effect on both apoptosis-sensitive and apoptosis-resistant cancer cells [6], whereas in human glioblastoma cells it is able to induce cell death, through a paraptosis-like mechanism [7].

Melanoma is a highly malignant tumour induced by transformation of melanocytes [8], whose incidence rate is rapidly increasing in the world [9]. Due to its high resistance to cytotoxic agents [10,11], metastatic melanoma has a very poor prognosis. Therefore, finding new anti-cancer molecules able to integrate or enhance chemical treatments of drug-resistant tumours such as melanoma is a relevant research issue.

In the present study we characterized the OP-A effects on A375 (BRAF V600E) and CHL-1 (BRAF wt) melanoma derived cell lines, as compared to the HaCaT (immortalised keratinocytes) cell line. To this purpose, we analysed cell viability, nuclear and mitochondria morphology and functionality, cell death induction, as well as cell cycle progression. Finally, we performed a comparative proteomic analysis on A375 cell line treated with OP-A.

## Materials and Methods

### Cell culture and treatments

A375 human melanoma cell line was grown in RPMI 1640 medium (Lonza, Switzerland) supplemented with 2 mM L-glutamine (Thermo Fisher Scientific, MA, USA), CHL-1 human melanoma and HaCaT immortalised human keratinocytes cell lines were grown in DMEM medium (Lonza), both supplemented with 10% Foetal Bovine Serum (FBS, Thermo Fisher Scientific), and penicillin/streptomycin (Sigma Aldrich, MO, USA) in an humidified 5% CO_2_ atmosphere at 37°C. Cell treatments: 1x10^5^ or 2x10^6^ cells were seeded in 12 wells plates or 100 mm dishes and the next day treated with the indicated amount of OP-A, diluted in fresh culture medium, for the indicated times. For the necrostatin treatments cells were incubated for 2 h with 20 μM necrostatin-1 (Santa Cruz Biotechnology, TX, USA) in complete medium, before the addition of OP-A.

### MTS viability assay

Cell viability was assessed by Acqueous One Solution Proliferation Assay (MTS assay, Promega, WI, USA), following the manufacturer indications.

### Western blotting

Whole cell extracts were prepared by lysis in RIPA buffer (50 mM Tris-HCl, pH 7.4, 150 mM NaCl, 0.5% Na-deoxycolate, 0.1% SDS, 1% NP-40, 2 mM Na_2-_EDTA), supplemented with protease inhibitors (Roche, Germany). Protein concentration was determined by Bio-Rad protein assay (Bio-Rad, CA, USA) [12] and 10–25 μg of proteins were separated on 4–12% Nu-PAGE pre-cast gels (Thermo Fisher Scientific). After blotting on PVDF and 1 h saturation in PBS containing 0.05% Tween-20 and 5% skim milk, membranes were incubated for 1 h or overnight with primary antibody, diluted in PBS containing 0.05% Tween-20 and 0.5% skim milk, washed three times for 10 min in PBS containing 0.05% Tween-20, incubated for 1 h with the appropriate horseradish peroxidase-conjugated secondary antibody (Bio-Rad) and the signals detected with Chemiglow by means of a FluorChem SP system (Alphainnotech, Germany). Primary antibodies were against: PARP, (BioMol, Germany, 1 μg/ml), Caspase 3 (9662, Cell Signaling, MA, USA, 1 μg/ml), Caspase 9 (9502, Cell Signaling, 1 μg/ml), LC3 (2775, Cell Signaling, 1 μg/ml), LC3B (D11 XP, Cell Signaling, 1 μg/ml), PINK1 (D8G3, Cell Signaling, 1 μg/ml), BAX (2D2 and N-20, Santa Cruz, 0.5 μg/ml), BAK (N-20, Santa Cruz, 0.5 μg/ml), cytochrome *c* (556432, Becton Dickinson, NJ, USA, 1 μg/ml). β-Tubulin (Sigma Aldrich, 1 μg/ml) was used as a loading control for cell extracts.

### Mitochondrial imaging, mitochondrial membrane potential, mitochondrial mass, lysosome contents, and mitochondrial reactive oxygen species (ROS) measurement

Mitochondrial network imaging was performed by incubating untreated and treated cells for 20 min at 37°C with 1 μM MitoTracker Red CMXRos regent (Thermo Fisher Scientific) in RPMI medium and nuclei counterstained with 1 μM HOECHST 33342 (Thermo Fisher Scientific). Images were captured by means of a Floid Instrument (Thermo Fisher Scientific).

For mitochondrial membrane potential, mitochondrial mass, lysosome content and mitochondrial ROS measurement, cells were detached with trypsin and, after washing with PBS, 1x10^6^ cells were incubated for 30 min at 37°C with 2 μM JC-1 (5,5’,6,6’-tetrachloro-1,1’,3,3’tetraethylbenzimidazolylcarbocyanine iodide), or 100 nM MitoTracker Green FM (Thermo Fisher Scientific), or 100 nM LysoTracker Red DND 99 (Thermo Fisher Scientific), or 5 μM MitoSOX Red (Thermo Fisher Scientific), diluted in pre-warmed complete medium. At the end of the incubation, samples were acquired by means of a FACSCalibur (Becton Dickinson).

### Flow cytometry analysis of cell death

Cells, untreated or treated with OP-A for the indicated times, were detached with trypsin, washed twice with PBS and after suspension in propidium iodide solution (50 μg/ml propidium iodide, 0,1% Triton X-100, 0,1% Na-citrate in PBS), incubated 8 h or overnight at 4°C in the dark. Samples were acquired by means of a FACSCalibur and flow cytometry data analysed with the FlowJo software (TreeStar, www.flowjo.com).

### 2D electrophoresis

Cells were treated with 0.6 μM OP-A for 24 h. After incubation, cells were detached with trypsin, washed twice with ice-cold PBS and centrifuged. Cell pellet was resuspended in Lysis Buffer (50 mM Tris-HCl pH 7.4, 1% Triton-X-100, 250 mM NaCl, 5 mM EDTA) and incubated overnight at 4°C. Cell lysates were centrifuged at 14,000 x g for 20 min and protein concentration in the supernatant was determined by Bradford assay. Equivalent protein amounts (300 μg) of control and treated cell samples were desalted by precipitation with cold ethanol (overnight at -20°C). Precipitates were centrifuged at 15,000 x g for 15 min. Protein pellets were dissolved in IEF buffer (9 M urea, 4% w/v CHAPS, 0.5% v/v Triton X-100, 20 mM DTT, 1% w/v Bio-Rad carrier ampholytes pH 3–10 NL). Protein concentration was estimated by using the Bradford assay, modified according to Ramagli and Rodriguez [13]. IPG strips (17 cm pH 3–10 NL, Bio-Rad ReadyStrip) were rehydrated overnight with IEF buffer containing 350 μg of total proteins. Proteins were focused using a Protean IEF Cell (Bio-Rad) at 12°C, by applying the following voltages: 250 V (90 min), 500 V (90 min), 1000 V (180 min) and 8000 V for a total of 52 KVh. After focusing, proteins were reduced by incubating the IPG strips with 1% w/v DTT in 10 ml of equilibration buffer (50 mM Tris-HCl pH 8.8, 6 M urea, 30% w/v glycerol, 2% w/v SDS and a dash of bromophenol blue), for 20 min, and then alkylated with 2.5% w/v iodoacetamide in 10 ml of equilibration buffer, for 20 min. Electrophoresis in the second dimension was carried out on 12% T polyacrylamide gels (180 x 240 x 1 mm) running on a Protean apparatus (Bio-Rad) in 25 mM Tris-HCl pH 8.3, 1.92 M glycine and 1% w/v SDS, with 120 V (for 12 h), until the dye front reached the bottom of the gel. 2-DE gels were then stained with colloidal Coomassie G250; resulting images were acquired by using a GS-800 imaging systems (Bio-Rad). For quantitative analysis, each biological sample was analyzed in technical triplicates.

### Gel image analysis

Digitalized images of Coomassie-stained gels were analyzed by using the PD Quest (vers. 7.3.1) 2-D analysis software (Bio-Rad), which allowed spot detection, landmarks identification, aligning/matching of spots within gels, quantification of matched spots and their analysis, according to manufacturer's instructions. Manual inspection of the spots was performed to verify the accuracy of automatic gel matching; any error in the automatic procedure was manually corrected prior to the final data analysis. The spot volume was used as the analysis parameter for quantifying protein expression. The protein spot volume was normalized to the spot volume of the entire gel (i.e., of all the protein spots). Fold-changes in protein spot levels were calculated between spot volumes in the treated group, relative to that in the control gels. Statistically significant changes in protein expression were determined by using two sequential data analysis criteria. First, a protein spot had to be present in all gels for each sample to be included in the analysis. Next, statistically significant changes in protein expression were determined by using the distribution of fold-change values in the data. Spots were determined to be statistically significant if the difference between the average intensity of a specific protein spot in the treated and control cells (three technical replicates of three biological samples) was greater than one standard deviation of the spot intensities for both groups. An absolute two-fold change in normalized spot densities was considered indicative of a differentially represented component; values 2 or 0.5 were associated with increased or decreased protein amounts after treatment, respectively.

### Protein digestion and mass spectrometry analysis

Spots from two-dimensional electrophoresis were manually excised from gels, triturated and washed with water. Proteins were in-gel reduced, S-alkylated and digested with trypsin, as previously reported [14]. Protein digests were subjected to a desalting/concentration step on microZipTipC18 devices (Millipore, Bedford, MA, USA). Peptide mixtures were then analyzed by nano-liquid chromatography coupled to electrospray-linear ion trap-tandem mass spectrometry (nanoLC-ESI-LIT-MS/MS) using a LTQ XL mass spectrometer (Thermo Fisher Scientific) equipped with Proxeon nanospray source connected to an Easy-nanoLC (Proxeon, Denmark) [15]. Peptide mixtures were separated on an Easy C18 column (100 x 0.075 mm, 3 μm) (Proxeon) using a gradient of acetonitrile containing 0.1% formic acid in aqueous 0.1% formic acid; acetonitrile ramped from 5% to 35% over 15 min, and from 35% to 95% over 2 min, at a flow rate of 300 nl/min. Spectra were acquired in the range m/z 400–2000. Acquisition was controlled by a data-dependent product ion scanning procedure over the three most abundant ions, enabling dynamic exclusion (repeat count 2 and exclusion duration 1 min). The mass isolation window and the collision energy were set to m/z 3 and 35%, respectively.

### Protein identification

MASCOT software package version 2.2.06 (Matrix Science, www.matrixscience.com) was used to identify proteins within spots from an updated human non-redundant sequence database (NCBI 2014/12). NanoLC-ESI-LIT-MS/MS data were searched by using a mass tolerance value of 2 Da for precursor ion and 0.8 Da for MS/MS fragments, trypsin as proteolytic enzyme, a missed cleavages maximum value of 2, and Cys carboxamidomethylation and Met oxidation as fixed and variable modification, respectively. Protein candidates with at least 2 assigned unique peptides with an individual MASCOT score >25, both corresponding to *p* < 0.05 for a significant identification, were further evaluated by the comparison with their calculated Mr and pI values, using the experimental ones obtained from two-dimensional electrophoresis.

### Data analysis

All the data reported were verified in at least six different replicates and are reported as mean ± SEM. Statistical analysis on flow cytometry data was performed by means of ANOVA and Bonferroni post-test. Analysis of gel spot quantitative differences was carried out using the Student's *t* test.

## Results

### OP-A induced cell death in human melanoma cells by activating the mitochondrial pathway of apoptosis

**Fig 1:**
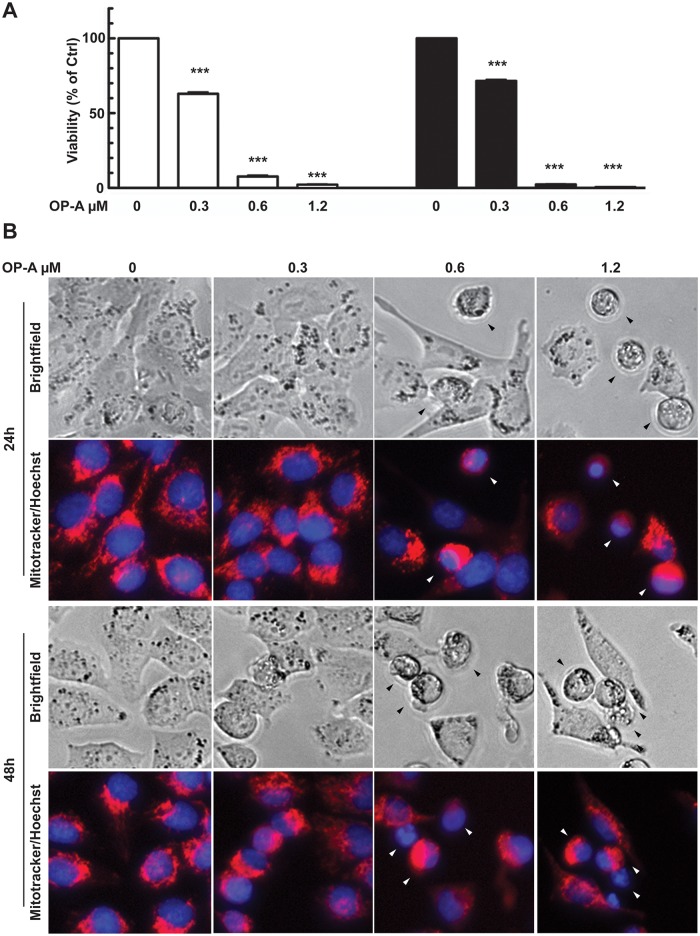
OP-A reduced A375 melanoma cells viability. Cells were incubated with 0.3, 0.6 and 1.2 μM OP-A for 24 and 48 h. Panel A: metabolic activity assayed by MTS test. Data are expressed as % of cell survival with respect to control. ***, p<0.0001. Panel B: mitochondrial and nuclear morphology. Mitochondrial network imaging was performed by incubating untreated and treated cells for 20 min at 37°C, with 1 μM MitoTracker Red CMXRos reagent in RPMI medium and nuclei counterstained with 1 μM HOECHST 33342. Images were captured by means of a Floid instrument. Arrowheads indicated picnotic and fragmented nuclei.

We first assessed the effect of OP-A on A375 human melanoma cell line, by analyzing cell viability by means of MTS assay in a dose-response treatment (0.3, 0.6, 1.2 μM OP-A) for 24 and 48 h. Fig 1A shows that administration with 0.3 μM OP-A for 24 h was effective in reducing cell metabolic activity at about 60% of control cells; metabolism was quite completely inhibited by 0.6 and 1.2 μM. The effect was slightly increased after 48 h administration. These results suggested that OP-A treatment resulted in an impairment of mitochondrial functionality, an effect that could lead to the induction of the intrinsic pathway of apoptosis. To verify this hypothesis, we then analyzed mitochondrial and nuclear morphology of cells treated as above using MitotrackerRed and Hoechst staining. Fig 1B shows that OP-A did not lead to the appearance of the cytoplasmic vacuolization observed during paraptosis induction [7], but resulted in a fragmentation of the mitochondrial network, even at the lowest dose used, and in the clustering of mitochondria. Moreover, we detected the appearance of picnotic and apoptotic nuclei (black and white arrowheads) when cells were treated with 0.6 and 1.2 μM OP-A.

**Fig 2:**
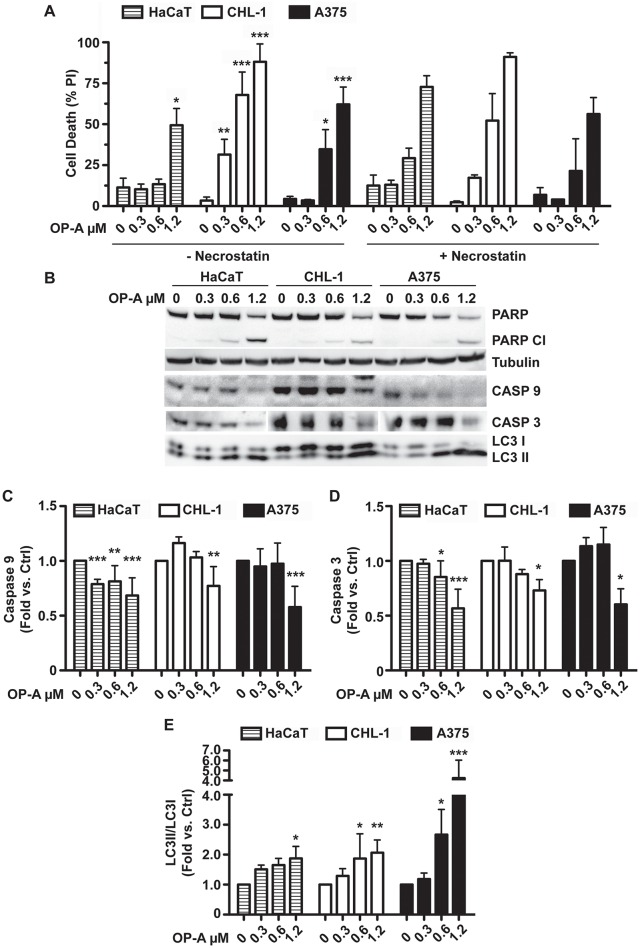
OP-A induced autophagy and cell death by apoptosis. A, flow cytometry analysis of cell death. HaCaT, CHL-1 and A375 cell lines were incubated with 0.3, 0.6 and 1.2 μM OP-A for 24 h and cell death analysis performed by propidium iodide staining and flow cytometry evaluation of the sub-G1 population. Where indicated, 20 μM necrostatin-1 was added to evaluate the occurrence of necrotic cell death. B, western blot of PARP, caspase-9 and -3, and LC3II proteins. C, D and E. Densitometric analysis of immunorecognized protein bands of caspase-3, caspase-9 and LC3II, respectively. Data were analysed with FlowJo software. *, p<0.05, **, p<0.01, ***, p<0.001.

Taken together, these results suggest that OP-A treatment of A375 cell line might lead to apoptosis induction through the activation of the mitochondrial pathway, and prompted us to expand our analysis to other melanomas and normal cell lines. To this purpose we compared OP-A-dependent cell death induction of A375 (BRAF V600E) with CHL-1 (BRAF wt) melanoma, and HaCaT (immortalised keratinocytes) cell lines. Fig 2A, shows that OP-A was able to induce cell death in the three cell lines. Nevertheless, treatment with 0.3 and 0.6 μM OP-A did not induce significant cell death levels in the HaCaT cell line, whereas these doses were effective in the two melanoma cell lines. At the highest dose tested (1.2 μM), OP-A caused massive cell death in all the three cell lines.

Western blot analysis of proteins involved in apoptosis (Fig 2B), revealed the activation of the mitochondrial apoptotic pathway [16]. In fact, we could detect PARP and caspase-9 and -3 cleavage (quantified in Fig 2C and 2D), respectively. In addition, we checked for the induction of autophagy using the specific marker LC3II, whose levels increase upon LC3I cleavage [17]. Fig 2B shows that OP-A induced autophagy in all the three cell lines, as demonstrated by the increase of LC3II/LC3I ratio (panel B). Densitometric analysis revealed that autophagy induction was particularly efficient in the A375 cell line (Fig 2E).

In order to exclude the possibility that other types of death could be responsible for the effects we observed, we performed the same OP-A treatments in presence of 20 μM necrostatin-1. Fig 2A, right panel, shows that necrostatin-1 did not inhibit OP-A-induced cell death, thus excluding the possible induction of necrosis or necroptosis [16].

**Fig 3:**
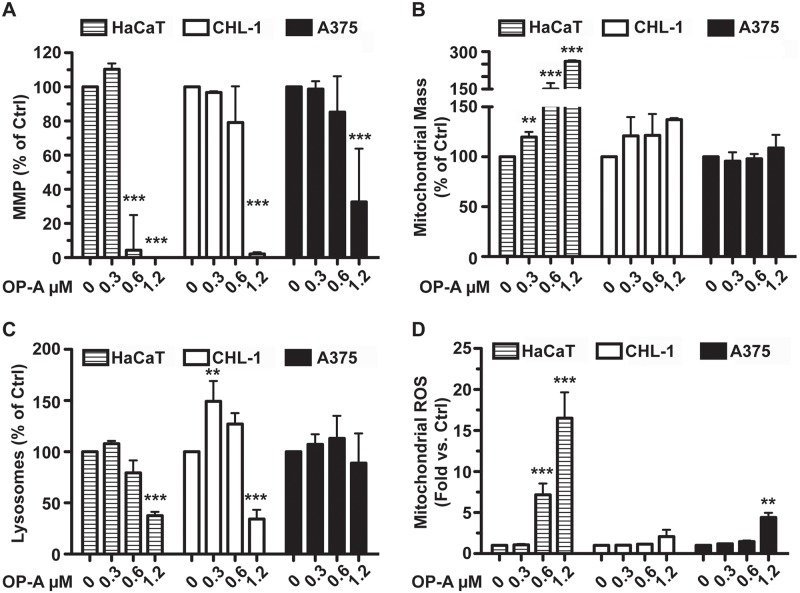
OP-A effect on mitochondrial membrane potential, mitochondrial mass, lysosome content and mitochondrial ROS production. HaCaT, CHL-1 and A375 cell lines incubated with 0.3, 0.6 and 1.2 μM OP-A for 24 h, were analysed to evaluate: A, mitochondrial membrane potential (MMP), (2 μM JC-1 staining); B, mitochondrial mass (100 nM MitoTracker Green staining); C, lysosome content (100 nM LysoTracker Red staining); D, mitochondrial ROS (5 μM MitoSox Red staining). Cells were incubated for 30 min at 37°C and then acquired by means of a FACSCalibur. Data, expressed as %, or fold increase with respect to controls, were analysed with FlowJo software. *, p<0.05, **, p<0.01, ***, p<0.001.

The OP-A-dependent mitochondrial perturbation we observed in the A375 cell line (Fig 1B) should be coupled not only to mitochondrial network rearrangements, but also to other alterations such as loss of mitochondrial membrane potential, which could result in mitophagy induction, and ROS generation. To verify these hypothesis, we first analysed mitochondrial membrane potential, mitochondrial mass, lysosome content and mitochondrial ROS production, by means of flow cytometry after staining with JC-1, MitoTracker Green, LysoTracker Red or MitoSOX Red, respectively. Fig 3A shows that OP-A treatment resulted in a reduction of mitochondrial membrane potential in all the three cell lines. As far as the HaCaT cell line, significant reduction of membrane potential occurred already at 0.6 μM OP-A, with a complete loss of the potential at 1.2 μM. On the other hand, the two melanoma cell lines showed a significant reduction of membrane potential only at 1.2 μM.

**Fig 4:**
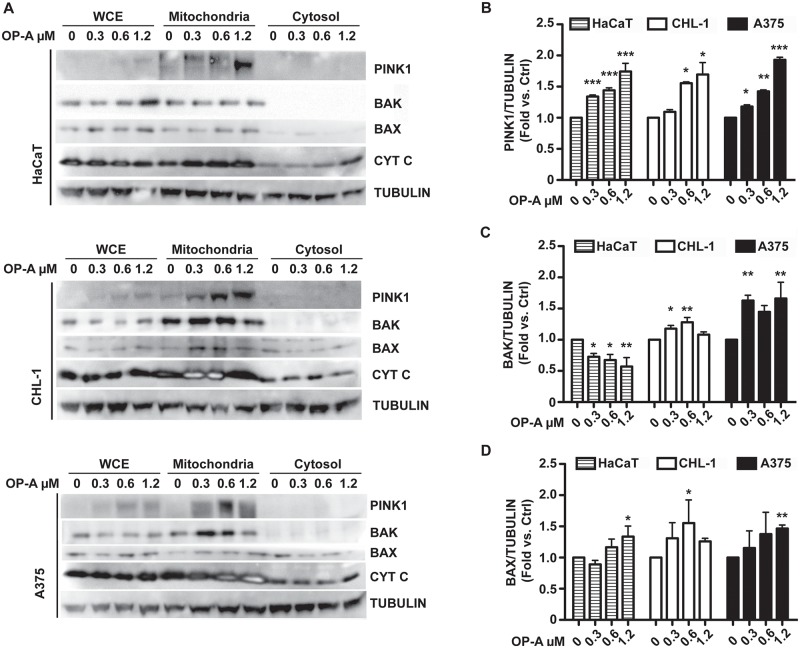
OP-A induced PINK1 accumulation, Bax and Bak translocation and cytochrome *c* release. HaCaT, CHL-1 and A375 cell lines were incubated with 0.3, 0.6 and 1.2 μM OP-A for 24 h and whole cell extracts (WCE), mitochondrial-enriched and cytosolic fractions were analysed by western blot (A) with antibodies directed against PINK1, Bak, Bax, and cytochrome *c*. Panels B, C and D, densitometric analysis of PINK1, Bak and Bax levels present in the mitochondrial fraction. β-tubulin was used as loading control and standard for densitometry. Data, expressed as %, or fold variation with respect to β-tubulin, were analysed with FlowJo software. *, p<0.05, **, p<0.01, ***, p<0.001.

Mitochondrial damages resulting in loss of membrane potential are frequently coupled to mitophagy induction, in order to remove the damaged organelles. For this reason, we measured mitochondrial mass (Fig 3B) and lysosome content (Fig 3C). Surprisingly, even if we observed significant induction of autophagy (Fig 2B and 2E) we were not able to detect any significant decrease of the mitochondrial mass, and for the HaCaT cell line we revealed a significant increase of the mass in samples treated with 0.6 and 1.2 μM OP-A. Nevertheless, we observed a significant reduction of the lysosome content in cells treated with 1.2 μM OP-A, thus suggesting autophagy (mitophagy) induction. We measured also mitochondrial-derived ROS, by means of MitoSOX Red. Fig 3D shows that OP-A induced ROS production in the HaCaT and A375 cell lines, while no significant ROS levels were detected for the CHL-1 cell line. Taken together these results suggest that OP-A damaged mitochondria, causing loss of mitochondrial membrane potential and ROS production, even though at different extents in the three cell lines. As far as the observed increase of mitochondrial mass, we could speculate that the reported effect of OP-A on the Endoplasmic Reticulum (ER) dilation coupled with alteration of the cell’s membranes permeability [7], could determine MitoTracker Green accumulation also in the ER, thus leading to an apparent increase in the mitochondrial mass. In order to confirm the occurrence of mitochondrial damage and induction of the mitochondrial pathway of apoptosis, we performed a western blot analysis on different cellular fractions of HaCaT, CHL-1 and A375 cell lines treated with OP-A for 24 h. Fig 4A shows the results of the analysis of PINK1, a marker of mitochondrial depolarization, Bax, Bak and cytochrome *c*, markers of the mitochondrial pathway of apoptosis [16].

OP-A induced significant PINK1 accumulation on the mitochondria in all the three cell lines (Fig 4A and 4B), thus confirming the mitochondrial damage and induction of mitophagy. Furthermore OP-A caused relocalisation to mitochondria of Bax and Bak proteins (Fig 4A, 4C and 4D), as well as, the release of cytochrome *c* in the cytosol (Fig 4A). The activation of the mitochondrial pathway of apoptosis in melanoma cell lines was also corroborated by the analyses of the autophagy marker LC3II (Fig 2B and 2E), which was increased in all the three cell lines upon OP-A treatment.

### OP-A induced alteration of the cell cycle

In order to test the effect of OP-A on cell cycle progression, we analysed the distribution of cells in each phase of the cycle after 24 h of OP-A administration, by means of flow cytometry after propidium iodide staining.

**Fig 5:**
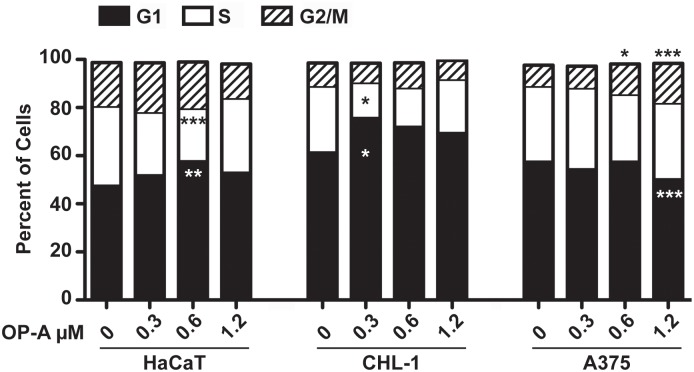
OP-A induced cell cycle alteration. HaCaT, CHL-1 and A375 cell lines, incubated with 0.3, 0.6 and 1.2 μM OP-A for 24 h, were stained with propidium iodide and, after over-night incubation at 4°C, the percentage of cells in each phase of the cycle measured by flow cytometry. *, p<0.05, **, p<0.01, ***, p<0.001.

As shown in Fig 5, OP-A induced alterations of the cell cycle in all cell line tested. As far as the HaCaT cell line, the only significant alteration was an increase of the percentage of cells in the G1 phase, from 47% (ctrl) to 57% (0.6 μM, p<0.01), coupled to a decrease of the percentage of cells in the S phase, from 33% (ctrl) to 22% (0.6 μM, p<0.01). A similar behaviour was observed for the CHL-1 cell lines, but at a lower dose. In fact, OP-A induced an increase of the percentage of cells in the G1 phase, from 61% (ctrl) to 75% (0.3 μM, p<0.05), coupled to a decrease of the percentage of cells in the S phase, from 28% (ctrl) to 15% (0.3 μM, p<0.05). Differently, in the A375 cell line, OP-A induced alteration of the cell cycle at the highest doses of the treatment. The percentage of cells in the G2/M phase, increased from 8% (ctrl) to 12% (0.6 μM, p<0.05) and to 12% (1.2 μM, p<0.001), coupled to a decrease of the percentage of cells in the G1 phase, from 57% (ctrl) to 50% (1.2 μM, p<0.001).

Taken together, our results suggest that prolonged exposure to OP-A leads to an impairment of mitochondrial functions, which are reflected in the alteration of cell cycle progression, induction of autophagy and ultimately in cell death by apoptosis, even if with different timings and modalities in the three cell lines.

### Effect of OP-A treatment on the protein repertoire of melanoma cells

**Fig 6:**
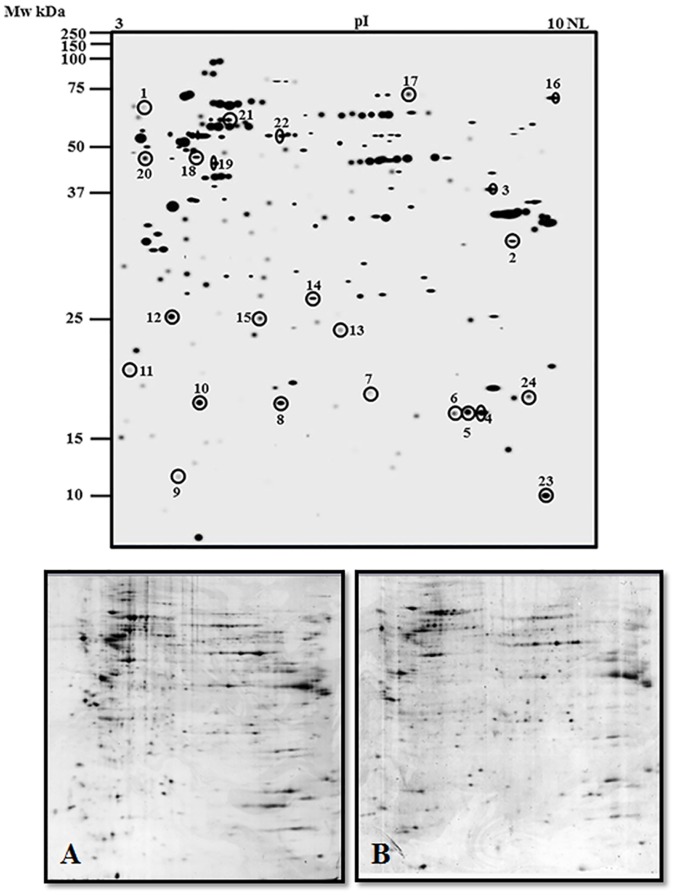
2-DE map of whole cell protein extract of OP-A-treated A375 melanoma cells. Proteins were extracted from cells treated with 0.6 μM OP-A for 24 h and from control cells. Proteins were electrophoretically separated in the non-linear pH range 3–10 and the 200–15 kDa molecular mass range, and visualized by colloidal Coomassie staining. Top panel: master gel. The encircled spots indicate the proteins affected by OP-A treatment, which were further subjected to nanoLC-ESI-LIT-MS/MS analysis. Data significance was evaluated by a Student’s t-test (*p*< 0.05). Bottom panel: representative gels of control (A) and OP-A treated (B) A375 melanoma cells.

In order to obtain information about the molecular mechanisms underlying OP-A effect, a proteomic analysis on the A375 cell line was carried out. Total proteins were extracted from control or 0.6 μM OP-A treated cells and resolved by two-dimensional gel electrophoresis (2-DE). To detect quantitative changes in relative protein spot volume, colloidal Coomassie-stained gels were subjected to software-assisted image analysis. Statistical evaluation of the relative volumes allowed to detect spots whose representation varied significantly (*p* < 0.05). The 2-D Master gel of the A375 human melanoma cell proteome is shown in Fig 6, upper panel, where encircled spots indicate differentially represented proteins.

The overall 2-DE profiles of control and treated cells were very similar; however, 24 protein spots whose abundance varied at least two-fold in response to OP-A challenge were detected. These differentially represented spots were excised from the gel, proteolyzed and subjected to MS analysis. Database searching with results from nanoLC-ESI-LIT-MS/MS experiments allowed the identification of the proteins migrating within these spots. The list of the identified polypeptides is reported in Table 1.

**Table 1:** Identification of proteins differentially represented in OP-A-treated and control A375 melanoma cells as revealed by 2-DE and nanoLC-ESI-LIT-MS/MS analysis. Spot numbering corresponds to that reported in Fig 6. Spot number, NCBI accession, protein name, MASCOT score, theoretical mass and pI values, peptides and unique peptides identified during analysis, sequence coverage, constitutive amino acids, emPAI score and fold change in treated cells with respect to control are shown.

Spots	Accession	Protein name	Mascot score	Theor. Mass	Theor pH	Peptides	Unique Peptides	Sequence coverage (%)	Lenght	emPAI	Fold change
**1**	gi|112910|sp|P02765.1	α-2-HS-glycoprotein	136	40098	5.43	3	3	5.4	367	0.2	2.0
	gi|187383|gb|AAA59553.1	Microtubule-associated protein 4	135	121618	5.35	3	3	3.6	1152	0.06	
	gi|117501|sp|P27797.1	Calreticulin	294	48283	4.29	7	7	22.1	417	0.35	
**2**	gi|130683|sp|P21796.2	Voltage-dependent anion-selective channel protein 1	1130	30868	8.62	23	18	73.5	283	10.32	0.2
**3**	gi|4557976|pdb|4ALD|A	Fructose 1,6-bisphosphate aldolase	847	39720	8.39	22	18	47.1	363	3.64	0.1
**4**	gi|30168|emb|CAA37039.1	Peptidylprolyl isomerase	563	18229	7.68	23	11	66.1	165	11.2	0.2
**5**	gi|30168|emb|CAA37039.1	Peptidylprolyl isomerase	498	18229	7.68	18	10	53.3	165	9.06	0.3
**6**	gi|30168|emb|CAA37039.1	Peptidylprolyl isomerase	559	18283	7.68	20	12	55.2	165	11.02	0.2
**7**	gi|6563248|gb|AAF17218.1	Prefoldin subunit 2	439	16695	6.2	21	10	70.8	154	4.34	0.2
**8**	gi|35595|emb|CAA77660.1	Pr22 protein	582	17292	5.76	35	15	70.5	149	15.87	0.4
**9**	gi|11907956|gb|AAG41412.1	P1725	181	10488	4.82	3	3	31.2	93	1.65	3.0
**10**	gi|181997|gb|AAA58453.1	Initiation factor 4D	524	17049	5.08	21	11	41.6	154	10.61	0.3
**11**	gi|5454028|sp|P10301.1	Ras-related protein R-ras	562	23155	6.44	15	14	80.1	218	5.65	2.0
	gi|30168|emb|CAA37039.1	Peptidylprolyl isomerase	515	18229	7.68	11	10	70.9	165	4.65	
	gi|181997|gb|AAA58453.1	Initiation factor 4D	108	17049	5.08	3	2	19.5	154	0.85	
**12**	gi|37496|emb|CAA34200.1	Translationally-controlled tumor protein isoform 2	504	19697	4.84	16	10	55.2	172	7.55	0.3
**13**	gi|999892|pdb|1HTI|A	Triosephosphate isomerase	935	26807	6.51	47	16	83.1	248	15.06	0.4
**14**	gi|51476543|emb|CAH18256.1	Heterogeneous nuclear ribonucleoprotein H1 (H), isoform CRA_b	279	21607	7.22	9	5	41.2	194	2.68	0.5
**15**	gi|37247|emb|CAA49379.1	Triosephosphate isomerase	566	26938	6.45	12	10	49.8	249	2.27	0.4
**16**	gi|32467|emb|CAA68445.1	71 kDa Heat shock cognate protein	302	71082	5.37	7	7	15.2	646	0.29	0.1
**17**	gi|119573383|gb|EAW52998.1	Lamin A/C, isoform CRA_c	741	87829	8.91	13	13	22.4	790	0.45	3.4
	gi|17158056|gb|AAA17976.2	FUSE binding protein	692	67690	7.18	15	14	28.1	644	1	
**18**	gi|16878077|gb|AAH17247.1	FUBP1 protein	637	68790	6.85	16	14	22.4	653	0.88	0.2
	gi|129379|sp|P10809.2	60 kDa Heat shock protein, mitochondrial	505	61187	5.7	12	10	22.9	573	0.8	
	gi|114549|sp|P06576.3	ATP synthase subunit beta, mitochondrial	741	56525	5.26	22	12	32.9	529	1.61	
	gi|3387929|gb|AAC28642.1	β-Tubulin	458	50095	4.78	11	9	21.6	444	1.05	
	gi|1710248|gb|AAB50217.1	Protein disulfide isomerase-related protein 5	656	46512	4.95	15	12	37.1	421	1.34	
	gi|1255188|gb|AAC50423.1	Dynamitin	406	44906	5.06	7	7	25.6	406	0.62	
**19**	gi|129379|sp|P10809.2	60 kDa Heat shock protein, mitochondrial	930	61187	5.7	20	18	36	573	1.89	0.2
**20**	gi|2809324|gb|AAB97725.1	Calumenin	633	37164	4.47	12	12	48.6	315	1.62	0.3
**21**	gi|292059|gb|AAA67526.1	MTHSP75	1193	74019	5.97	36	21	34.2	679	2.56	2.2
	gi|193788318|dbj|BAG53212.1	Heat shock cognate 71 kDa protein isoform 1	496	68166	5.32	13	12	20.6	621	0.7	
	gi|1051170|gb|AAB07787.1	GAP SH3 binding protein	421	52189	5.36	11	8	21.7	466	0.74	
	gi|241478|gb|AAB20770.1	Heterogeneous nuclear ribonucleoprotein complex K	610	51230	5.39	14	12	29.8	463	1.17	
**22**	gi|220702506|pdb|3F8U|A	Tapasin chain A, ERP57 heterodimer	1418	54541	5.61	50	28	63	481	3.89	0.4
**23**	gi|27900298|emb|CAD61608.1	10 kDa Heat shock protein, mitochondrial	761	10924	9.22	58	18	90.2	102	14.36	0.1
**24**	gi|127983|sp|P22392.1	Nucleoside diphosphate kinase B	342	17401	8.52	12	7	51.3	152	5.08	0.1
	gi|469171|gb|AAA50953.1	Chaperonin 10	214	10925	8.89	18	6	60.8	102	0.87	

Globally, spots assayed were associated with 16 non-redundant protein entries. Analysis of spot 1, 11, 17, 18, 21 and 24 resulted in a multiple identification, and they were not further discussed. One protein, peptidyl prolyl isomerase was present in multiple spots (4–6) whose structural differences were not further characterized; probably, they resulted from post-translational modifications or sequence-related isozymes. Fifteen proteins resulted down-regulated, whereas 1 was up-regulated. Functional categorization according to Gene Ontology annotation and literature data (data not shown), showed that differentially-represented proteins grouped into different functional categories, including components involved in glucose metabolism, protein folding, mitochondrial transport, cytoskeleton organization and cell proliferation.

Reprogramming of metabolism, involving enhanced glycolysis is a hallmark of cancer [18]. Hence, down-regulation of glycolytic enzymes can contribute to reduce energy fuelling to cancer cells. Two enzymes of the glycolytic metabolism have been identified, namely fructose 1,6 bisphosphate aldolase A [19] (ALDOA, spot 3) and triose phosphate isomerase (TPI, spots 13 and 15). Notably, ALDOA is highly expressed in a variety of malignant cancers, including renal cancer [20], human lung squamous cell carcinoma [21,22], and hepatocellular carcinomas [23], suggesting it could promote cancer growth by enhancing glycolysis. It is worth to note that OP-A has been described as a calmodulin inhibitor [24] and that calmodulin antagonists induce a reduction of ALDOA levels, coupled to cell death in melanoma cell lines [25].

TPI catalyzes the conversion of dihydroxyacetone phosphate into D-glyceraldehyde 3-phosphate, a key substrate of the glycolytic pathway. This enzyme is up-regulated in different types of lung and urinary cancers [26,27] and in ovarian carcinoma [28]. In etoposide-treated HeLa cells, inhibition of TPI by cyclin A/Cdk2 phosphorylation hampers energy production, thereby inducing apoptosis [29]. Interestingly, a recent body of evidence supports the idea that glycolytic enzymes play also non-glycolytic roles, which are essential for promoting cancer cell proliferation and chemoresistance [16].

Six proteins involved in protein folding were down-regulated by OP-A, namely peptidyl-prolyl cis-trans isomerase A (spots 4–6), prefoldin (spot 7), heat shock protein (HSPA8) (spot 16), mitochondrial 60 kDa and 10 kDa heat shock proteins (spot 19, 23), and tapasin (spot 22). Peptidyl-prolyl cis-trans isomerases A (PPIase A) [30] belongs to the cyclophilins class of PPIases. Recently, evidence of the involvement of members of this family in cancer development has been reported. In fact, knockdown of cyclophylin A reverses chemoresistance in endometrial cancer cells [31], while the over-expression of peptidyl-prolyl isomerase-like 1 is associated with growth of colon cancer cells [32]. Prefoldins (PFDs) [33] are hetero-oligomer chaperones involved in cancer development, since their main targets are actin and tubulin [33]. Up-regulation of members of the PFD protein family has been reported in different tumours, such as glioblastoma [34], breast [35], pancreatic [36], colon [37], bladder [38] and cervical [39] carcinomas. The heat shock proteins (HSPs) [40] have been reported to be significantly elevated in many kind of human cancers and their over-expression has been correlated to therapeutic resistance and poor survival. HSP71 (HSPA8, spot 16) is a constitutively expressed isoform of the HSP70 protein family, essential for normal protein homeostasis in unstressed cells [41]. In addition to facilitating folding, HSC70 is involved in the degradation of misfolded proteins [42,43]. Although HSC70 is over-expressed in cancer cells, little is known about how it contributes to their survival. A recent proteomic investigation of human colon cancer cells showed that HSC70 interacts with and prevents the degradation of key proteins involved in cancer survival [44]. As far as mitochondrial HSPs isoforms (spot 19, 23), they can enhance tumourigenicity by promoting the retention into the mitochondria of pro-apoptotic factors [45].

Mitochondrial voltage-dependent anion channel 1 (VDAC-1, spot 2) is the most abundant and physiologically relevant isoform of the VDAC family of pore-forming proteins, located at the outer membrane of mitochondria (OMM) [46]. VDACs are voltage-sensitive ion channels that regulate the energy flux across the OMM by facilitating the diffusion of key molecules such as nucleotides, pyruvate and malate [46]. Altering VDAC-1 levels may lead to opposite effects in tumour cells. In fact, VDAC-1 favours the release of pro-apoptotic proteins from the mitochondrial inter-membrane space to the cytosol [47]. On the other hand, VDAC-1 sustains the high glycolytic rates of tumour cells by associating with hexokinase, and inhibits the formation of the mitochondrial permeability transition pore (MPTP) in the OMM, which is necessary for the release of pro-apoptotic factors [48]. Recently, a systematic analysis demonstrated that VDAC-1 is up regulated in breast, colon, liver, lung, pancreatic, and thyroid tumour tissues [49]. VDAC-1 is considered a marker of mitochondrial mass, and its decrease in OP-A treated A375 cells fairly correlates with the mitochondrial damage and reduction of mitochondrial mass due to mitophagy induction.

Three proteins involved in cytoskeleton dynamics regulation have been found as differentially represented after treatment with OP-A, namely Pr22 protein (spot 8) and calumenin (spot 20) (both down-represented), and P1725 protein (spot 9) (over-represented). Microtubule destabilization is a crucial process during cancer progression. Pr22 protein is over-expressed in different highly malignant tumours, such as breast or ovarian cancers and leukaemias [50–52], whereas its reduction can reverse the malignant phenotype [52]. Calumenin [53] is involved in the regulation of cytoskeleton and localization of intracellular proteins [54]. In a recent proteomic study concerning the effect of the anti-tumoural natural drug gambogic acid on breast carcinoma cells, levels of calumenin were consistently decreased [55].

Other proteins with different functions were observed as down-represented after treatment with OP-A. Among that, translationally-controlled tumour protein (TCTP, spot 12) that is an ubiquitous eukaryotic component involved in pivotal cell functions like growth, cell cycle, apoptosis and stem cells pluripotency [56]. TCPT is over-represented in diverse tumours, and its down-regulation hampers tumour viability [57]. Recent data indicate that TCTP has a chaperone-like structure that makes it able to interact with anti-apoptotic proteins like Bcl-xL, thereby functioning as a pro-survival factor in cancer cells [58].

## Discussion

In this study, we analysed the effect of OP-A on A375 (BRAF V600E) and CHL-1 (BRAF wt) human melanoma cell lines, as compared to the HaCaT immortalised keratinocytes cell line. In fact, despite a growing body of evidence indicates that OPs possesses anti-proliferative activity towards different cancer-derived cell lines, the specific mechanism underlying this action remains unclear.

Our results demonstrated that OP-A induced mitochondrial network fragmentation, membrane potential dissipation and mitochondrial ROS production, leading to induction of autophagy and ultimately resulting in activation of the mitochondrial pathway of apoptosis. Although flow cytometry analysis did not allow to show any significant reduction of mitochondrial mass, probably due to dye accumulation also in swelled ER, evidence of mithocondrial damage was obtained by western blot analysis. In fact, the strong increase in the mitochondrial levels of PINK1 protein, a marker of mitochondrial membrane potential depolarization, and of the autophagy marker LC3II, strongly put forward the occurrence of mitochondrial damage and induction of autophagy. A further piece of evidence resulted from proteomic investigation of A375 cell line, in which a dramatic reduction of the mitochondrial mass marker VDAC-1 was observed.

Mitochondrial damage and consequent mitophagy ultimately resulted in cell death by the activation of mitochondrial pathway of apoptosis, as indicated by the analysis of specific markers.

In fact, western blot analysis demonstrated caspase-9 and -3 activation, PARP cleavage, as well as relocalisation of the pro-apoptotic members of the Bcl-2 family Bax and Bak. The activation of the mitochondrial pathway of apoptosis was further supported by cytochrome *c* release in the cytosol.

Prolonged exposure to OP-A leads to an impairment of mitochondrial functions, induction of autophagy and ultimately in cell death by apoptosis in both melanoma cell lines, even though with different timings and modalities in different cell lines. In particular, CHL-1 cells appeared more sensitive to OP-A. This difference could be related to survival signalling pathways activated by the BRAF V600E mutation present in the A375 cell line.

Furthermore, the proteomic approach on A375 cell line allowed us to identify down-regulation of different proteins, essential to maintain cell integrity and viability, whose levels normally increase in different types of cancer.

Taken together, our results demonstrate that OP-A possess a strong cytotoxic activity on melanoma cells, even on recalcitrant A375 cell line, where it is able to induce mitochondrial damage and cell death by apoptosis. Under this respect, it is noteworthy that OP-A is effective at nanomolar/micromolar concentrations after 24 h of treatment, whereas the BRAF inhibitor vemurafenib, the elective drug for treatment of recalcitrant melanomas is not (data not shown). However, the mechanism of action of the toxin deserves further investigation. In fact, even though mitochondria appear as the primary site of action, the apparent lack of mitochondrial mass reduction and the proteomic identification of many down-regulated proteins regulating fundamental cellular processes suggest that other cell death mechanisms may be involved, such as for instance the ER stress.

In conclusion, even further investigation is needed, OP-A appears as a promising molecule to be studied for its potential use in the treatment of melanoma, possibly in association to other type of anti-cancer agents.
